# The Orthanc Ecosystem for Medical Imaging

**DOI:** 10.1007/s10278-018-0082-y

**Published:** 2018-05-03

**Authors:** Sébastien Jodogne

**Affiliations:** Scientific Collaborator, Montefiore Institute, B28, Department of Electrical Engineering and Computer Science, University of Liège, B-4000 Liège, Belgium

**Keywords:** DICOM, Vendor neutral archive, Teleradiology

## Abstract

This paper reviews the components of Orthanc, a free and open-source, highly versatile ecosystem for medical imaging. At the core of the Orthanc ecosystem, the Orthanc server is a lightweight vendor neutral archive that provides PACS managers with a powerful environment to automate and optimize the imaging flows that are very specific to each hospital. The Orthanc server can be extended with plugins that provide solutions for teleradiology, digital pathology, or enterprise-ready databases. It is shown how software developers and research engineers can easily develop external software or Web portals dealing with medical images, with minimal knowledge of the DICOM standard, thanks to the advanced programming interface of the Orthanc server. The paper concludes by introducing the Stone of Orthanc, an innovative toolkit for the cross-platform rendering of medical images.

## Introduction

Medical imaging has grown over years as an essential step for the diagnosis and treatment of many diseases in the clinical routine. By combining information arising from different imaging modalities (CT, PET, MRI, US…), patients benefit from extremely personalized, high-quality treatments [1]. Consequently, hospitals are getting equipped with a steadily growing number of imaging devices, which in turn results in an explosion of the volume of medical images that are generated. The analysis of such an amount of data implies the development and deployment of many specialized clinical software dedicated to specific fields of expertise. Simultaneously, as healthcare is moving to a patient-centric approach [2], the seamless and secure diffusion of medical images over Internet becomes an important concern: Lifetime links with the personal EHR (Electronic Health Record), continuity of care (moves of the patient, rare diseases…), pooling of imaging devices between several hospitals, or remote second opinions must be taken into consideration.

Such concerns about the volume, the processing, and the diffusion of medical images are also present within research centers and university hospitals. Indeed, both pre-clinical and clinical research is increasingly interested by big-data algorithms applied to the (semi-)automatic analysis of multimodal medical images [3]. Industrializing the software resulting from such a research is a complex task, as it must be able to smoothly integrate with the imaging infrastructure of hospitals. Similarly, sharing anonymized imaging datasets becomes crucial for educational purpose, for pharmaceutical research and as a support to open-science. All these factors explain why the data analysis of clinical images, the careful definition of the radiology workflow, as well as and the administration of the computer network of hospitals and research centers imply many technical challenges.

DICOM (Digital Imaging and Communications in Medicine) is the de facto standard for the encoding, management, and exchange of medical images [4]. It was conceived to provide best interoperability across hardware designed by different manufacturers. Each DICOM file (known as an *instance*) can be thought of as an envelope that embeds the medical image together with the clinical meta-data associated with this image. Meta-data is essentially encoded as a hierarchy of key-value pairs that is similar in spirit to the more recent XML or JSON file formats. Thanks to the DICOM network protocol (one of the earliest examples of Web services), the DICOM instances can hopefully be shared between the modalities generating these medical images, the central PACS server (Picture Archiving and Communication System) archiving all these images in the long-term, and the computers displaying or processing the images.

Unfortunately, several practical problems are often encountered if dealing with the DICOM standard. Indeed, even though the DICOM standard enables hardware compatibility spanning almost the whole medical imaging scope, commercial PACS solutions are mostly focused on radiology workflow in hospital setups. This tends to exclude some specific specialties (such as radiotherapy, nuclear medicine, digital pathology, dentistry, neuro-imaging, quality control, or intraoperative imaging) from professional PACS support. In such specialties, it is common to deal with software that does not support DICOM networking and/or that is not endorsed by the PACS managers of the hospital for security reasons. In practice, this leads to the emergence of manual, parallel, sometimes unofficial workflow based upon the export of images to CD/DVD burners, USB sticks, or network drives. This manual approach should obviously be discouraged, as it breaks the traceability of clinical data, as it might lead to security breaches, and as it makes such process tedious and error-prone (any CT or MRI scan results in hundreds of files without meaningful filenames that must be handled as a whole).

Furthermore, despite its universality, the DICOM format and its associated network protocol are complex and require a long learning curve, both for imaging practitioners, for network administrators and for research engineers. Because DICOM was not designed to facilitate the software manipulation and analysis of imaging data, many companies or researchers creating new image analysis software encounter compatibility issues while integrating with other manufacturers. A typical example is that of image compression: Even though DICOM does include built-in support for lossless compression through JPEG-LS or JPEG-2000, many software dedicated to medical imaging does not support such compression algorithms, which often leads hospitals to simply choose to avoid any compression, putting a high load on their network infrastructure. As a reaction to bypass the complexity of DICOM, some medical specialisms have also developed their own file formats (notably NIfTI and BIDS for neuro-imaging, or STL-like 3D models for dentistry). Unfortunately, such competing formats necessitate seamless integration with DICOM to be useful in clinical setups, for which few software currently exists.

Summarizing, each and every hospital or research center in the world needs custom, ancillary software to add more flexibility to the star-shaped DICOM network topology revolving around their radiology-focused PACS server. This flexibility is needed to tackle specialized processes that are parallel to the baseline radiology, notably: Adding imaging buffers between clinical departments, quick and easy anonymization, supplying images from the PACS to specialized or research software, Web publication of images, automation through scripting (e.g., to prefetch images or to monitor doses), and exchanges between hospitals (or with pharmaceutical firms for clinical research). Currently, such deployments are often made by highly skilled PACS managers who learn on the job, writing custom applications on the top of advanced free and open-source toolkits such as GDCM [5] or DCMTK [6], with sparse sharing of the technical knowledge between hospitals.

This paper describes the free and open-source Orthanc ecosystem, whose development was triggered in 2012 by these observations [7]. The long-term goal of the Orthanc project is to foster cooperation and knowledge sharing about medical imaging workflow in hospitals (including with vendors of imaging devices and data-science companies), to stimulate the development of new algorithms dedicated to multimodal medical imaging, as well as to improve interoperability in healthcare by providing a DICOM server that can be easily and quickly deployed at multiple places in hospitals as a complement to the main PACS server.

## Review

**Fig. 1:**
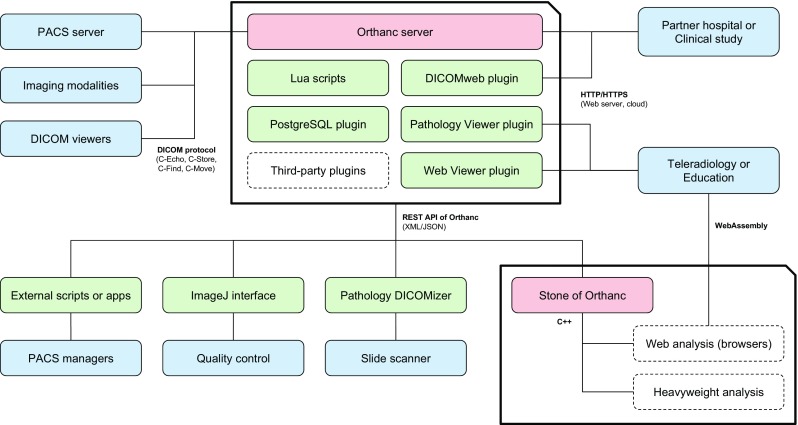
An overview of the entire Orthanc ecosystem. The two main components of Orthanc are depicted in red. Orthanc plugins as well as interfaces to external systems are depicted in green. Applications relevant to medical practice, academic activities, or clinical research are highlighted in blue

The Orthanc ecosystem is depicted on Fig. 1. In this section, we will successively review each of the components of this ecosystem.

### Orthanc Server

**Fig. 2:**
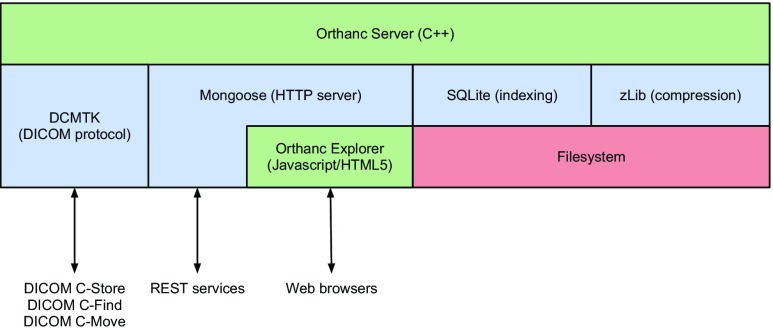
The layered software architecture of the Orthanc server. The embedded third-party components are depicted in blue. Note that Orthanc can act both as a service class provider (SCP) and a service class user (SCU) for the C-Echo, C-Find, C-Store, and C-Move DICOM commands

The core component of this ecosystem is the *Orthanc server*. The Orthanc server is inherently a VNA (Vendor Neutral Archive) that can receive, store, index, and transmit medical images according to the DICOM standard. Figure 2 depicts the internal architecture of the Orthanc server, that comes with the following desirable properties:

- Small footprint: It can run on any kind of hardware platform, from Raspberry Pi to large-scale cloud infrastructures by way of virtual machines or desktop computers. It is even possible to run the Orthanc server from a USB stick.
- Cross-platform: Entirely written in pure C++, it can be readily packaged for many operating systems. Binary packages for Microsoft Windows, Apple OS X, Docker, and several GNU/Linux or UNIX distributions (notably Debian, Fedora, and FreeBSD) are available.
- Standalone: It comes bundled with its own database engine (SQLite), which avoids the burden of configuring a database server for simple use cases. Similarly, no external software or framework (such as Java or .NET) is needed to run the Orthanc server.
- Compliant: Best DICOM interoperability is provided by embedding the well-known DCMTK toolkit [6]. Emphasis is also put on the quality and the automated validation of its source code.

Thanks to this attention to the simplicity of packaging, deploying an Orthanc server is almost immediate: In most cases, it is sufficient to download the binaries and to adapt a handful of options (such as the DICOM AET— Application Entity Title— of the server) by editing the provided configuration file. Interfacing an Orthanc server with peripheral imaging modalities, including the PACS of the hospital, amounts to declaring them in the configuration file by providing their respective DICOM parameters (AET, IP address, and TCP port number).

### Orthanc Explorer

Besides its built-in embedded DICOM server, the Orthanc server also comes bundled with an embedded HTTP server. This HTTP server exposes an embedded Web user interface that is known as *Orthanc Explorer*. This user interface allows the Orthanc users to browse and interact with the content of the DICOM store, from any client computer within the hospital network by using the default Web browser^1^. Obviously, the Orthanc server can be configured to use HTTPS and to assign passwords to users, in order to secure the access to the medical images through proper authentication and encryption.

**Fig. 3:**
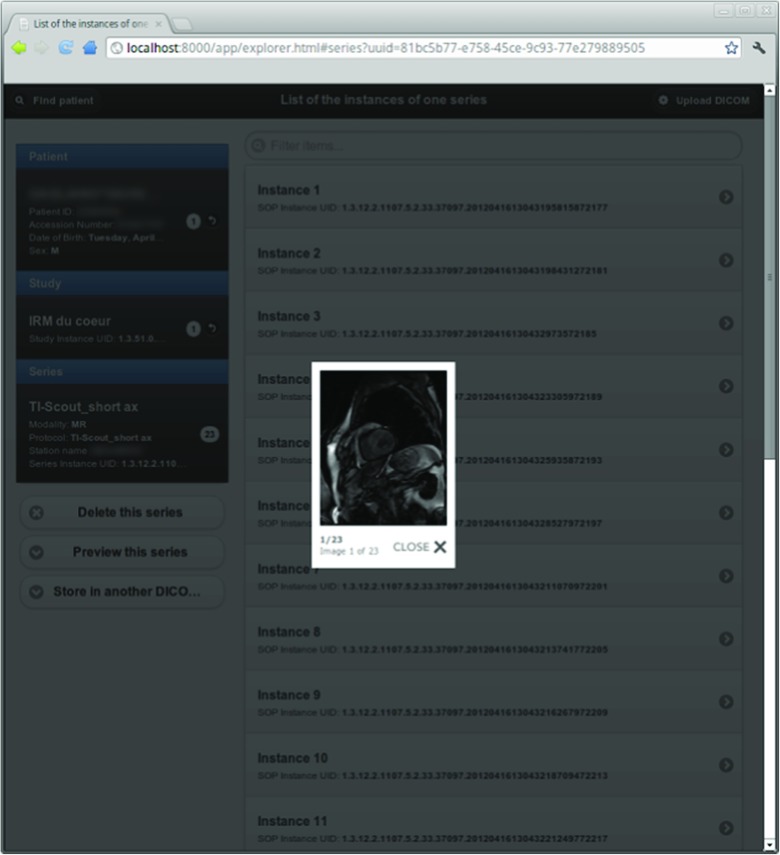
Previewing a DICOM series of images using Orthanc Explorer. This Web user interface displays the content of its hosting Orthanc server by browsing it according to the Patient/Study/Series/Instance DICOM model of the real world: The list of patients is first displayed, then one can successively open the studies of the patient, the series of the studies, and finally the instances of the series

**Fig. 4:**
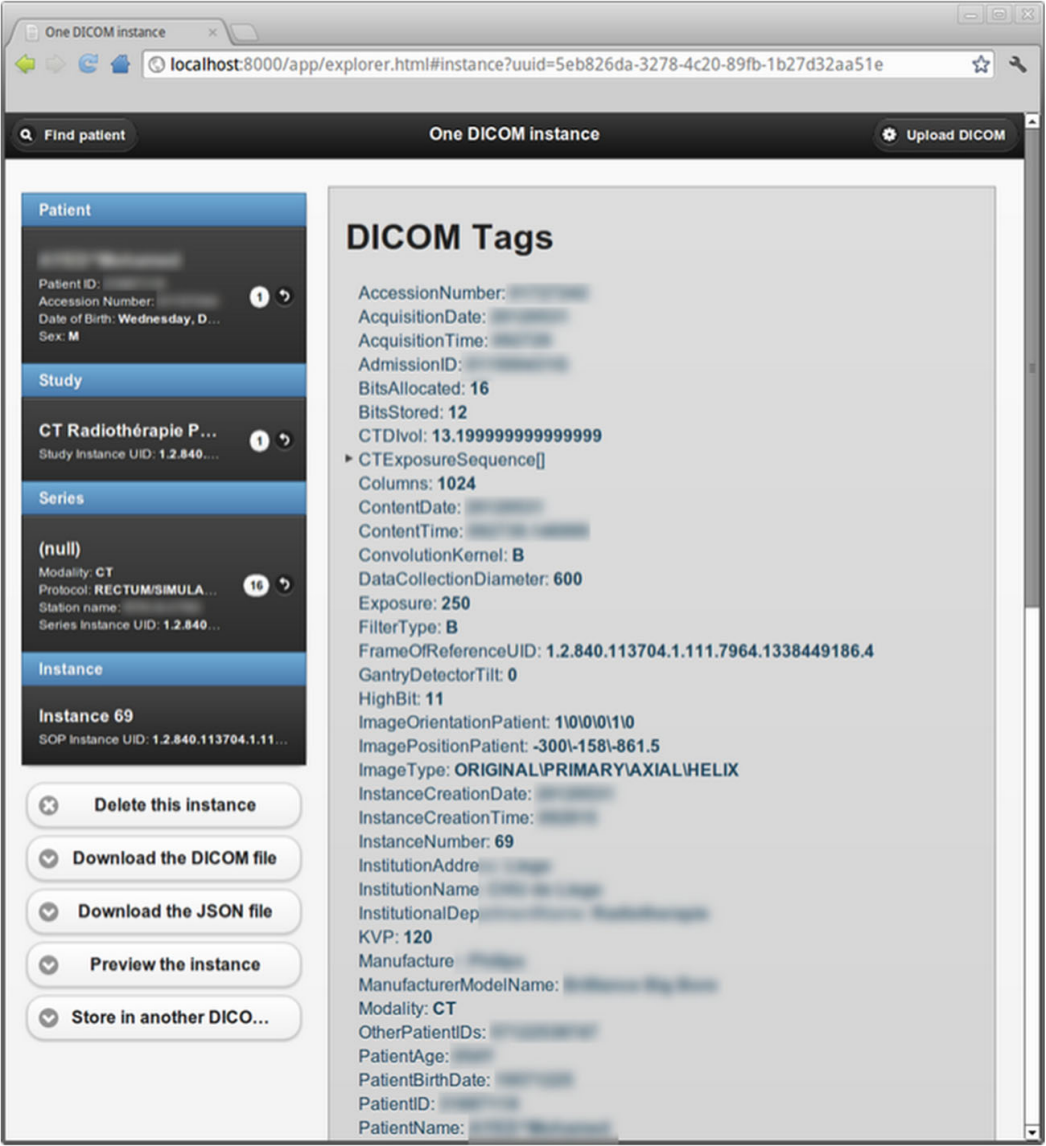
Displaying one DICOM instance with Orthanc Explorer. The right column displays the hierarchy of the DICOM tags. The bottom of the left column contains the action buttons

Figures 3 and 4 illustrate Orthanc Explorer in action. Besides browsing the content of the Orthanc server, this Web interface also allows to trigger actions on the server, such as anonymization, query/retrieve from remote modalities, download of ZIP archives, or manual upload of DICOM instances.

Thanks to Orthanc Explorer, adding a cohort of Orthanc servers to the DICOM topology of the hospital can already facilitate the data management for clinical routine and medical research by providing easy-to-install, task-specific, fine-grained DICOM stores. Such servers can act as interconnection bridges between DICOM modalities, between medical departments, yet even between hospitals. It is for instance used at the University Hospital of Liège in order to provide medical physicists of the radiotherapy department with a backup access to the contours produced by the nuclear medicine department^2^, and with a research database that collects in-room images (CBCT and radiographs) produced by the treatment machines [7].

### Lua Scripting

**Fig. 5:**
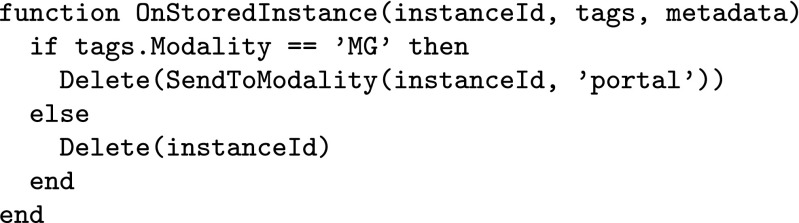
Sample Lua script that is executed for each DICOM instance received by the Orthanc server. This script routes the incoming images whose DICOM tag “Modality” (0008,0060) contains the “MG” value, to another DICOM modality whose symbolic name is “portal”. This script could typically be used to redirect mammography images acquired in a mobile X-ray unit to some radiology office

Orthanc Explorer enables the manual administration of Orthanc servers. In order to drive DICOM flows in an automated way, the Orthanc server also embeds a scripting engine that can be used to define routing rules using the Lua language. A Lua script can monitor the arrival of DICOM instances and react accordingly, for instance by redirecting them to another modality or by modifying them if some condition is met. For instance, Fig. 5 shows a sample Lua script that implements an auto-routing scenario in a few lines of code that are self-explaining and easily maintainable.

### REST API

Lua scripting is very useful for basic auto-routing tasks. For more advanced scenarios of DICOM automation, the Orthanc server provides a full programmatic access to all its core features, under the form of a REST API [8, 9] that is served through its embedded HTTP server. Thanks to this REST API, external scripts or applications can drive the Orthanc server, independently of the programming language that is used to develop them (such as Python, C#, Java, Matlab…). By delegating the handling of the DICOM file format and network protocol to the Orthanc server, it is possible to speed up the development of new applications dedicated to medical imaging (may they be heavyweight, mobile or Web applications). Indeed, the Orthanc server essentially acts as a high-level bridge between on one hand, the DICOM standard, and on the other hand, software standards that are well-known to every modern developer (such as JSON, XML, PNG, or HTTP).

**Fig. 6:**
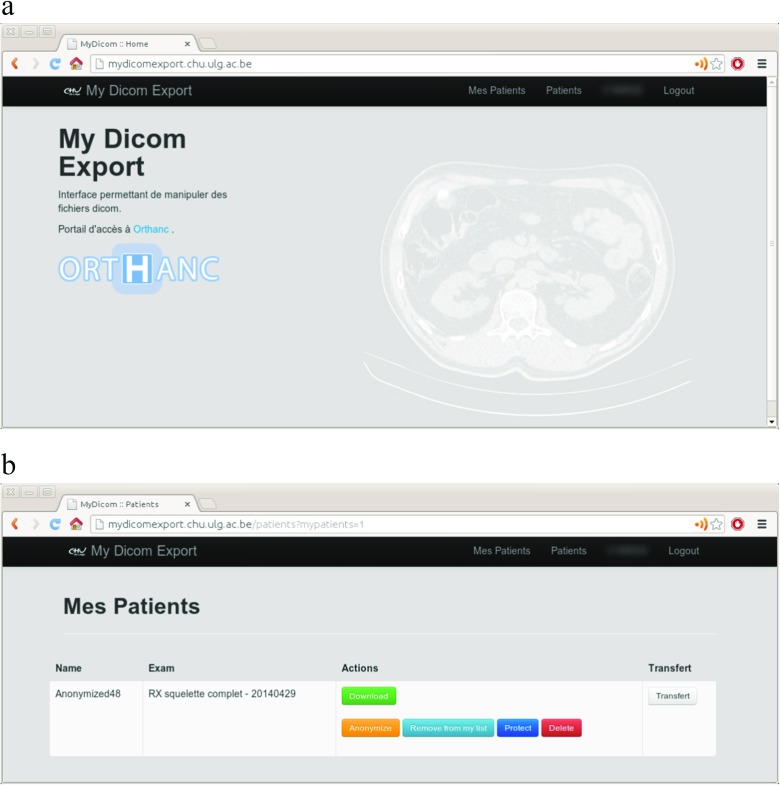
Two screenshots of the MyDicomExport application from the University Hospital of Liège that is dedicated to academic uses (research and education)

**Fig. 7:**
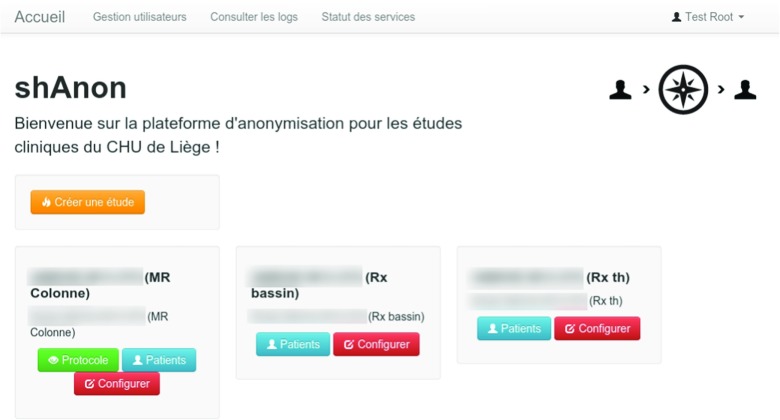
Screenshot of the shAnon application from the University Hospital of Liège that is dedicated to pharmaceutical research

**Fig. 8:**
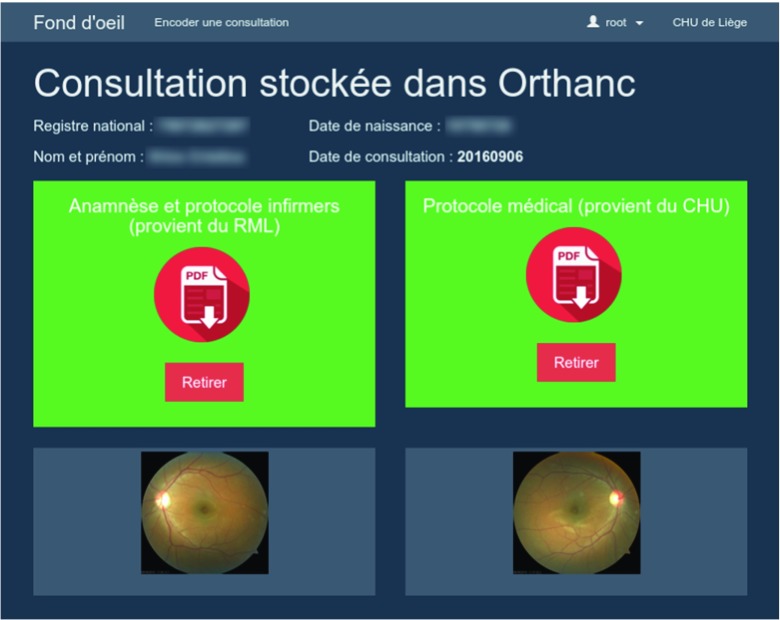
Screenshot of a Web platform dedicated to ophthalmology. Nurses working in remote offices can send fundus photographies to ophthalmologists working at the University Hospital of Liège

**Fig. 9:**
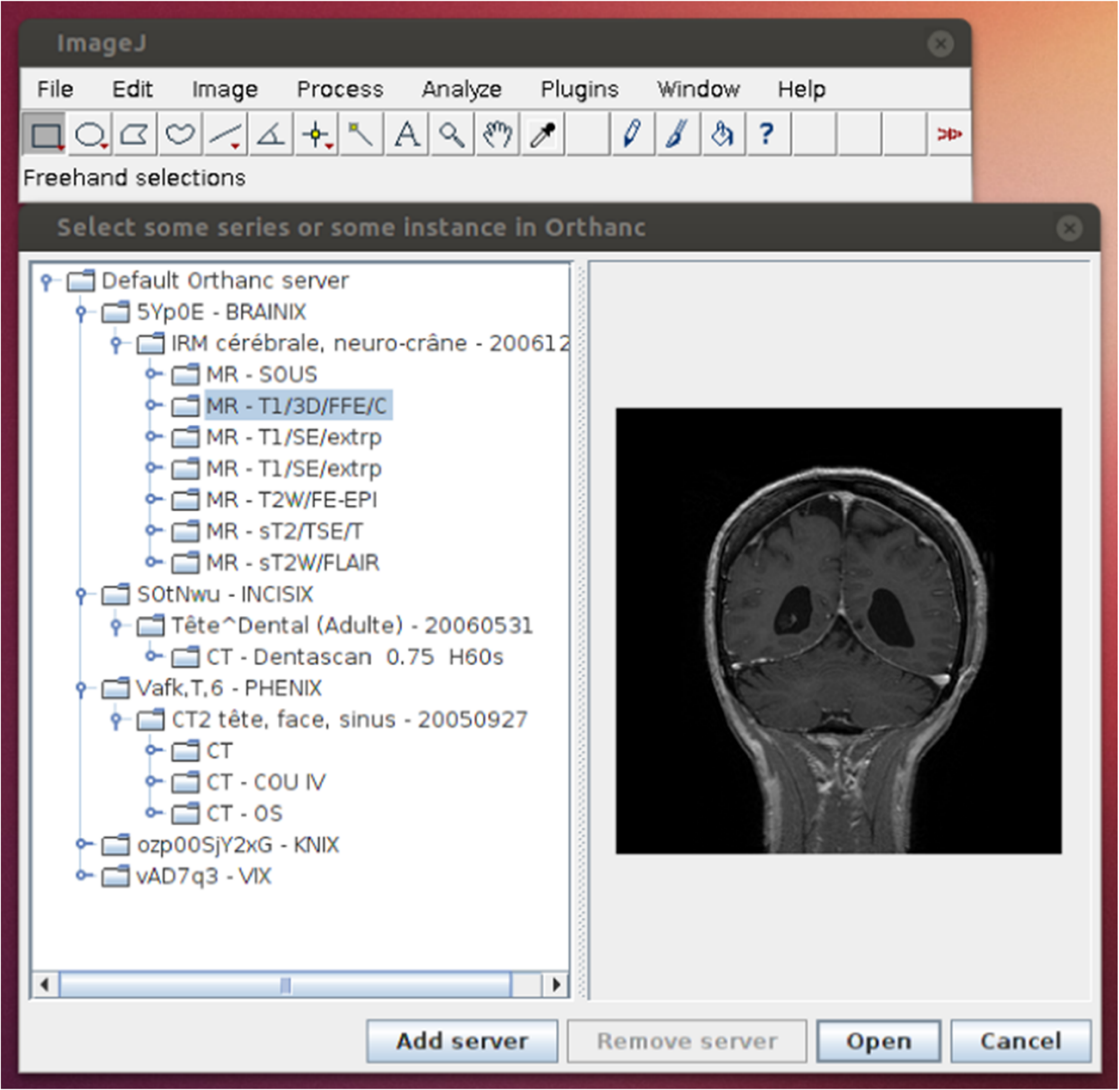
ImageJ interface to the Orthanc server. This interface greatly simplifies the access to DICOM images when dealing with ImageJ (e.g., for quality control of DICOM modalities, or for pedagogical use)

Technically, the Orthanc Explorer interface described in “Orthanc Explorer” is one example of an application that entirely relies on the REST API of the Orthanc server. Besides Orthanc Explorer, several applications have been built on the top of this REST API at the University Hospital of Liège. For instance, Fig. 6 illustrates one Web application deployed in the Intranet of the hospital that allows physicians, physicists, and veterinarians to download ZIP archives containing anonymized images extracted from the PACS, using any browser. This application is used for research, quality assurance, and educational purpose. Figure 7 depicts another Web application that automates the process of uploading anonymized images to external FTP servers managed by pharmaceutical firms or CROs (Clinical Research Organizations) in the context of clinical research making use of medical images. As another example, a Web platform dedicated to the remote follow-up of diabetes is illustrated in Fig. 8. As a last example of a clinical application built using the REST API, the Orthanc project provides an interface for ImageJ to remotely browse the content of an Orthanc server, then import 2D/3D DICOM images from it (cf. Fig. 9).

Research software has also been built using the REST API, with applications to the quantification of focal fibrosis in cardiac MRI, to the quality control of nuclear medicine modalities [10], to the comparison of delineation algorithms in oncology [11], or to the texture analysis of FDG PET/CT images [12].

It is very important to notice that the REST API of the Orthanc server can be used to securely transmit images between two remote sites over Internet, through the encrypted HTTPS protocol. This feature is used by hospitals in the Liège area (Belgium) to exchange their imaging data on a daily basis, particularly in the context of oncology. The Osimis S.A. company, spin-off of the University Hospital of Liège, was created alongside the Orthanc ecosystem in order to provide commercial services on the top of free and open-source project, notably to setup and maintain such inter-hospital links.

### Orthanc Plugins

**Fig. 10:**
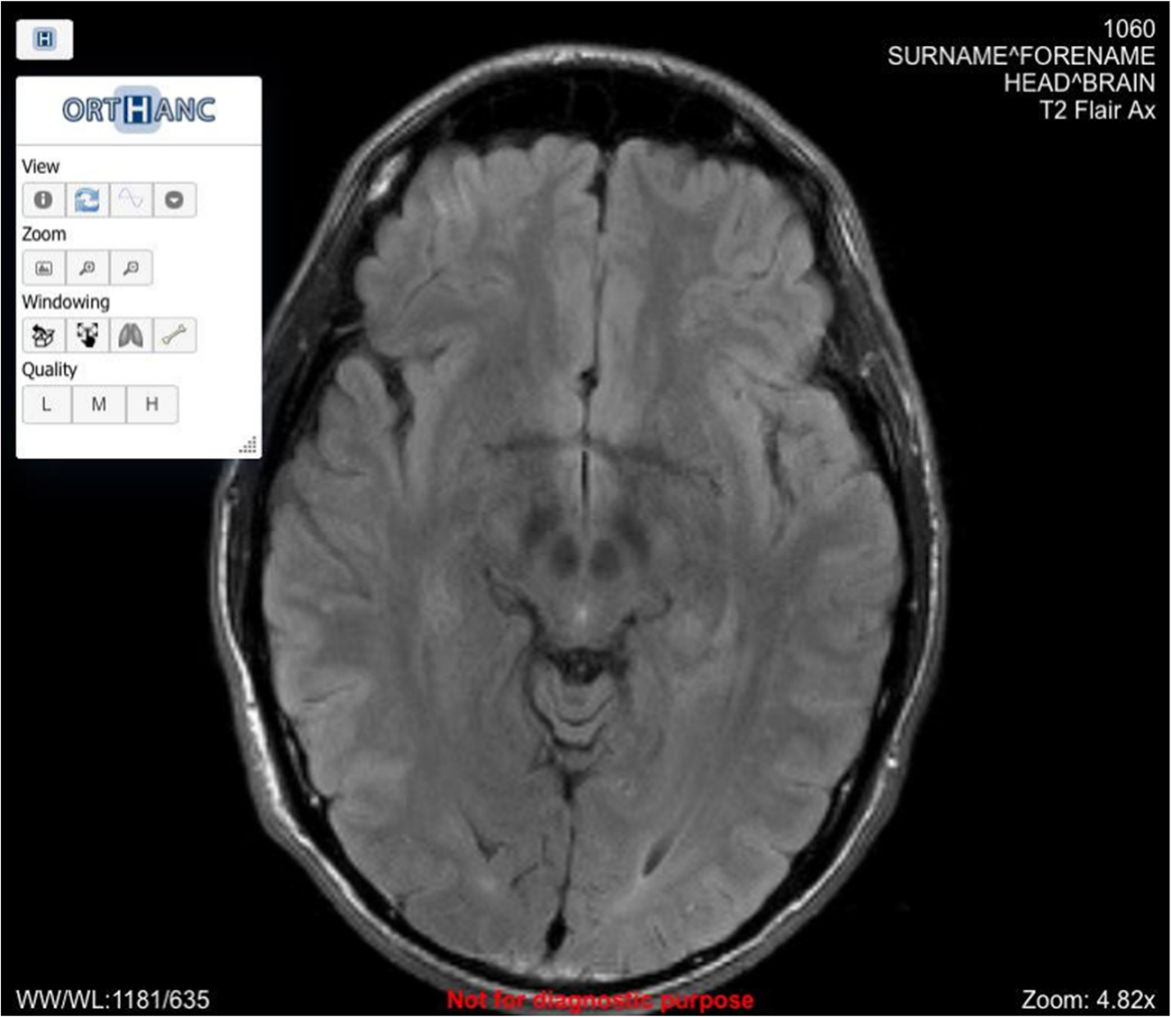
Screenshot of the official Web viewer plugin of the Orthanc project

**Fig. 11:**
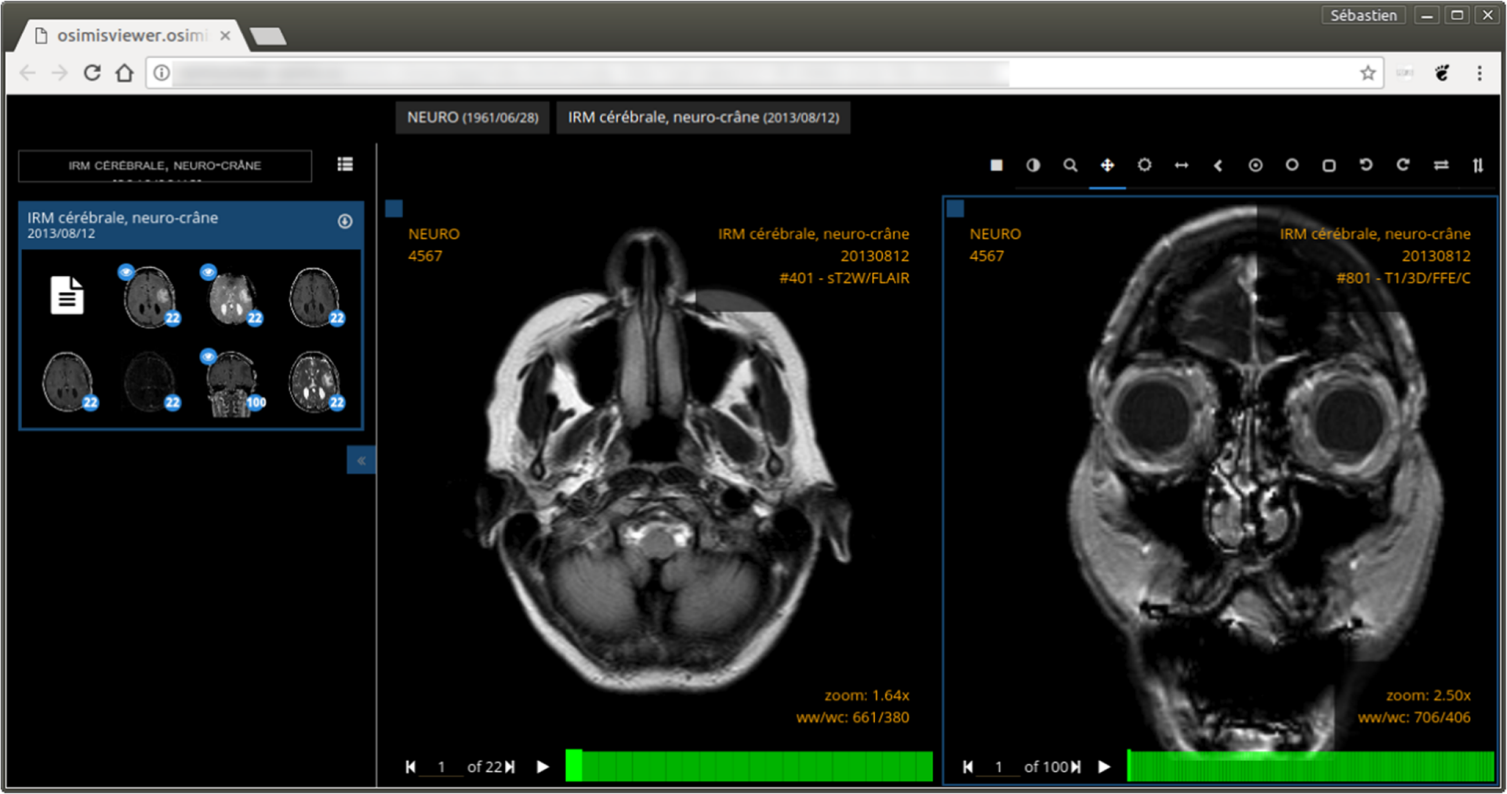
Screenshot of the advanced Web viewer plugin that is developed by Osimis S.A. as a separate free and open-source project on the top of the official Web viewer of the Orthanc project

Besides its Lua engine and its REST API, the core of the Orthanc server can also be extended through plugins. Such plugins are coded in C or C++, and can be used to serve new Web pages or to add new endpoints in the REST API. The most prominent example of an Orthanc plugin is the official Web viewer, that can be used to display series of DICOM images within a Web browser, as depicted in Fig. 10. This Web viewer plugin can be used to meet basic teleradiology needs. Osimis S.A. has enhanced this basic Web viewer with more advanced features (such as multi-series rendering, measurements, and CE labeling). A screenshot of the Osimis Web viewer is available as Fig. 11.

The plugin engine of Orthanc can also be used to replace the built-in SQLite database (that is suitable to store up to about 50,000 DICOM instances), by an enterprise-ready database (that can store a virtually unlimited number of DICOM instances). In that respect, the Orthanc project proposes a free and open-source plugin for PostgreSQL that has been reported to successfully manage at least 10TB of imaging data.

As depicted in Fig. 1, the Orthanc ecosystem also features a free and open-source plugin that proposes a reference implementation of DICOMweb, the emergent Web standard for medical imaging. It is also possible to serve DICOM worklists by developing a dedicated Orthanc plugin. The full list of Orthanc plugins is part of the Orthanc Book [13].

### Digital Pathology

**Fig. 12:**
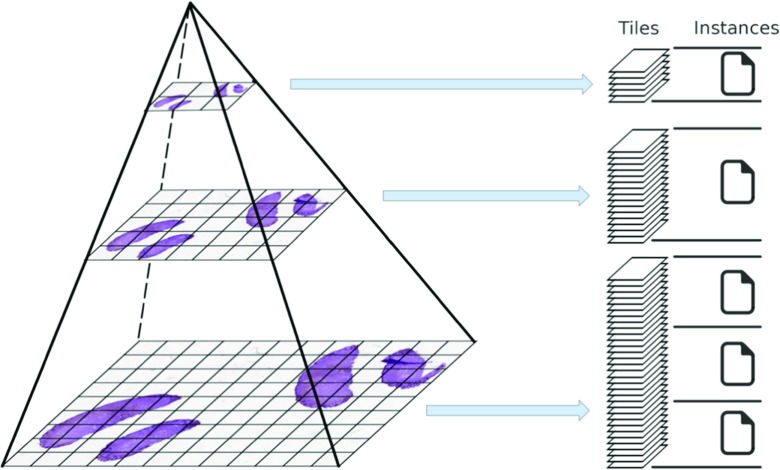
Mapping a multi-resolution pyramid according to the DICOM standard [14]. Each level of the pyramid is a downscaled version of the whole-slide image, and is decomposed as a set of tiles. The tiles are encoded as separate frames of multi-frame DICOM instances

**Fig. 13:**
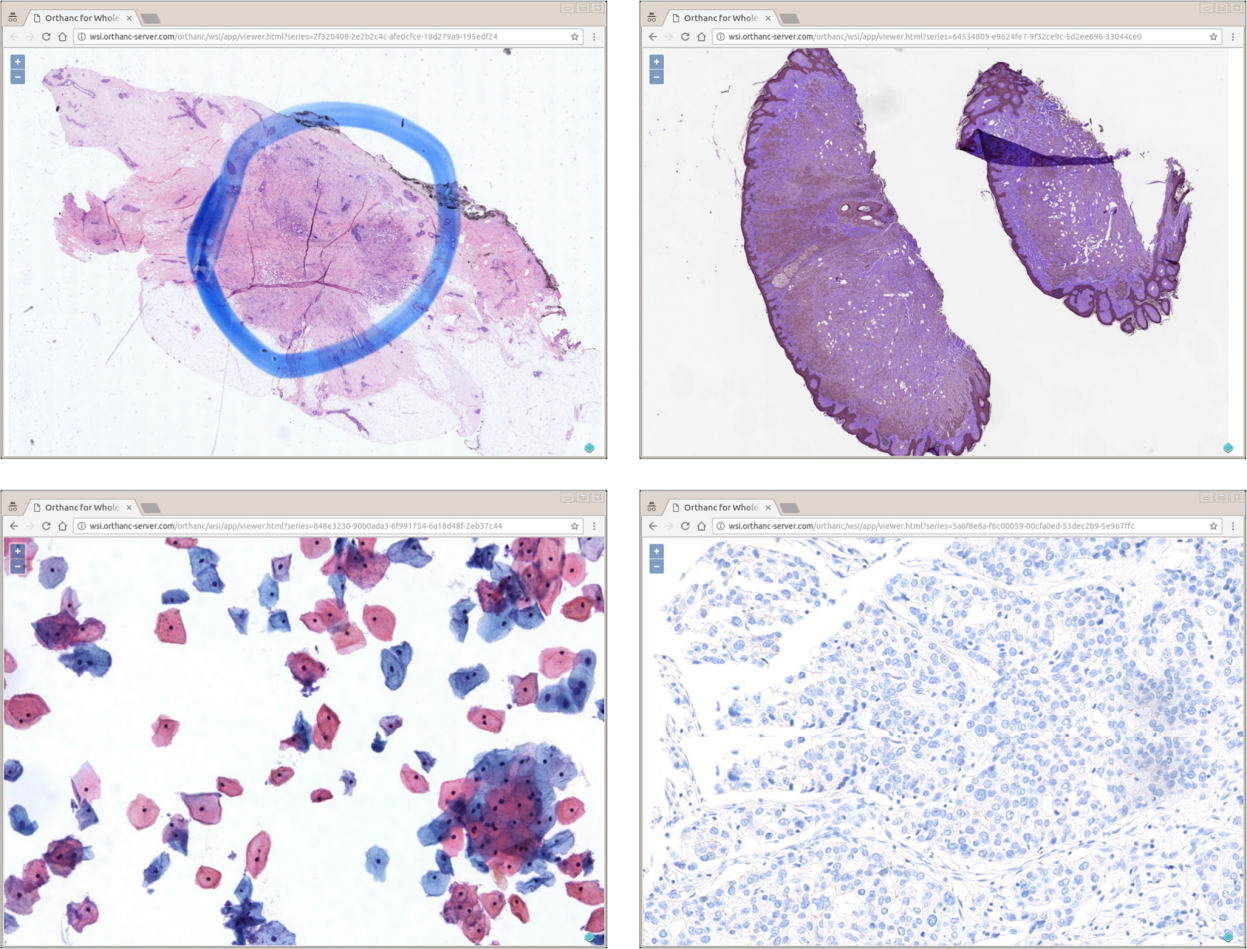
Some screenshots of a Web browser displaying real-world pathology DICOM images stored inside the Orthanc server, at various zoom levels. The Web application is zero-footprint: It is entirely written in pure JavaScript, and no heavyweight client must be installed (the rendering is entirely done by the Web browser). The Web interface is built upon OpenLayers version 3, a free and open-source JavaScript library for displaying raster tile maps [15]

Importantly, the Orthanc ecosystem has recently introduced free and open-source support of DICOM for digital pathology [16]. This opens the path to telepathology, an application of telemedicine that allows the practice of the anatomopathology over a long distance with the use of images in an electronic format rather than viewing glass slides [17]. Telepathology is potentially useful for intraoperative consultation [18], secondary consultation from experts [19], education, and research [20], and pathology archiving [21]. Figure 1 shows that the Orthanc framework for digital pathology and telepathology is divided into two separate components:

- The *DICOMizer*, a standalone command-line tool that takes as input a non-DICOM whole-slide microscopic image, and that generates a compliant DICOM file. Because digital pathology images are extremely large (typically a size of 100,000×100,000 pixels in RGB colors), the DICOM standard specifies how to encode such images as a multiresolution pyramid, a technique that is commonly used in geospatial Web mapping systems (cf. Fig. 12).
- A plugin for the Orthanc server that can serve such images over Internet using any Web browser. Screenshots of this plugin are provided in Fig. 13.

To the best of our knowledge, this software is the first open, reference implementation of DICOM for whole-slide microscopic imaging. We argue that spreading DICOM support for digital pathology is very important, as it enables closing the gap between clinical laboratories and hospitals by providing an unified exchange standard.

### Stone of Orthanc

The last component of the Orthanc ecosystem of Fig. 1 that has not been discussed yet is the Stone of Orthanc. Stone of Orthanc is a building block for the rendering of 2D/3D medical images. It notably features support for MPR (multiplanar reconstruction of volume images), reslicing, layering (fusion of images), radiotherapy (rendering of RT-DOSE and RT-STRUCT), and accurate physical 3D world coordinates. Obviously, it can retrieve DICOM images from an Orthanc server through its REST API.

**Fig. 14:**
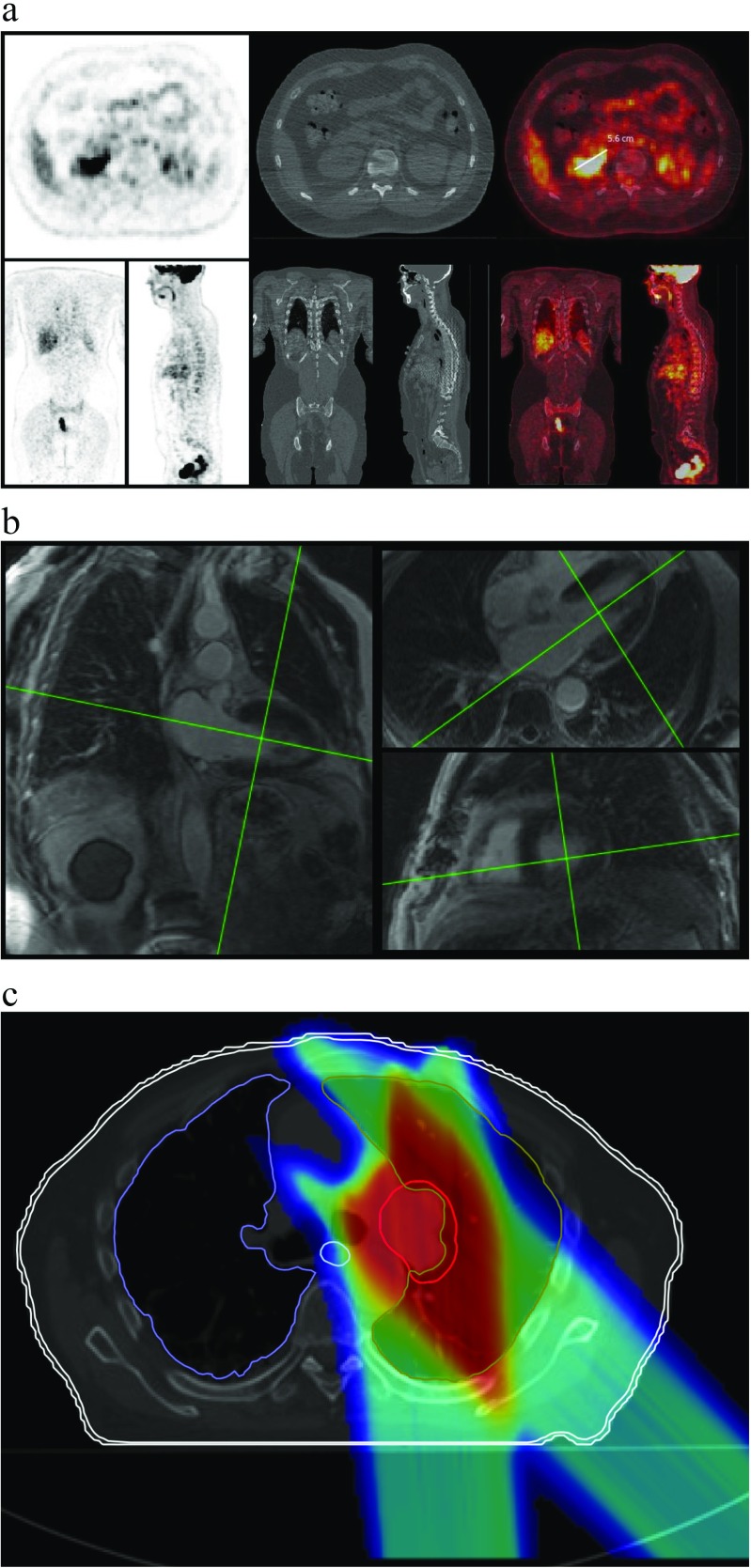
Three rendering of medical images produced by the Stone of Orthanc toolkit: **a** PET-CT fusion with axial, coronal, and sagittal projections, **b** multiplanar cardiac MRI, and **c** superimposition of radiotherapy doses and contours (RT-DOSE and RT-STRUCT) over a simulation CT

Stone of Orthanc takes the form of a lightweight, cross-platform C++ toolkit. As Stone of Orthanc is entirely written in pure C++ and is entirely standalone, it can easily be embedded into heavyweight software (C++ support comes out of the box, and bindings to Java and C# are in active development) or into native mobile applications (Android and iOS). Very importantly, the Stone of Orthanc is compatible with the emerging WebAssembly technology that can be used to run C++ applications in Web browsers without installing any browser extension [22]. As a consequence, thanks to the Stone of Orthanc, it is possible to benefit from a single codebase to quickly write applications for displaying and/or analyzing medical images, and targeting any kind of platform (native, mobile or Web). Figure 14 shows some rendering produced by the Stone of Orthanc toolkit.

## Discussion

Summarizing the previous review, Orthanc is a rich, integrated, evolutive ecosystem spanning a large portion of the medical imaging scope. As a free and open-source project, Orthanc is supported by a vibrant worldwide community of users that happily share their experience about medical imaging in clinical or research contexts. Corporate services (such as support, custom development, integration, maintenance, training...) are also currently available from Osimis S.A., who works according to an open-source business model. Other service companies on the top of the Orthanc ecosystem will hopefully show up in the future.

The Orthanc server is similar to DCM4CHEE [23]. Contrarily to Orthanc, however, DCM4CHEE gets into position as a full replacement to a large-scale PACS server, with advanced mechanisms to integrate with hospital information systems (HL7 messages, audit record repository, IHE cross-enterprise document sharing…). This contrasts with the Orthanc server that is rather developed as a *complement* to some large-scale radiology PACS in order to automate DICOM flows through scripting, or to facilitate the Web diffusion of images. It is possible that such enterprise-level features will be brought to Orthanc in the future, but they are not readily available at the time of writing. Also note that the Orthanc server can already act as a PACS for small-sized or medium-sized hospitals, community health centers or research centers, provided it is complemented by a certified DICOM viewer.

On the other hand, the Orthanc server is easier to install and configure than DCM4CHEE. Learning to use the Orthanc server requires almost no prerequisite (in particular, there is no need to setup a database server). Given its small footprint, many instances of the Orthanc server can be deployed in a hospital in order to smooth a set of individual, task-specific processes. Rich applications can be built on the top of the Orthanc server by driving its REST API. The Orthanc ecosystem is also focused on the interface with Web technologies.

As far as the Stone of Orthanc is concerned, it is very similar in spirit to Kitware’s VTK, a C++ toolkit for scientific visualization [24]. However, contrarily to VTK that entirely relies on the GPU (graphics processing unit) and that can be used in any field of scientific visualization, Stone of Orthanc is entirely focused on the CPU-based rendering of medical images. This makes Stone a highly cross-platform framework: A codebase written with Stone can run even on low-performance platforms that are not equipped with a GPU, as well as directly inside Web browsers. Stone of Orthanc is also related to Cornerstone, a client-side JavaScript toolkit to render medical images in Web browsers [25]. Contrarily to Cornerstone, Stone of Orthanc is written in C++. As a consequence, the latter can also be embedded into native or heavyweight applications, and readily benefit from the robust data analysis toolkits that are available for C++.

## Summary

This paper has described the Orthanc ecosystem for medical imaging [7]. Orthanc is used worldwide for applications such as the Web diffusion of (possibly anonymized) medical images, the automated exchange or routing of DICOM instances (both inside or outside of hospitals and research centers), the scientific research or industrial R&D about new imaging modalities or software, or the education of stakeholders (physicians or medical physicists).

At the core of this ecosystem lies the Orthanc server, a lightweight, novel, robust DICOM store. The Orthanc server features rich scripting capabilities (through its Lua engine and its REST API) and built-in compatibility with Web technologies, making it very versatile. The Orthanc server can be considered as a micro-service, in the sense of SOA (Service-Oriented Architectures): Higher-level external applications (such as Web portals) written in any language can handle DICOM by supervising an instance of Orthanc through its REST API. The Orthanc server is cross-platform and fully standalone, making its deployment quick and easy: In particular, numerous instances of the Orthanc server can be deployed inside one hospital to automate task-specific DICOM flows as a complement to the central PACS server.

Many plugins revolve around the Orthanc server. They can be used to reinforce the core features of Orthanc, by improving the user interface, by adding support for enterprise-ready databases (e.g., PostgreSQL), by interfacing with administrative servers of the hospital (e.g., supplying worklists from RIS through HL7 messages), by providing teleradiology solutions, or by implementing recent additions to the DICOM standard (such as DICOMweb or digital pathology support). The Orthanc ecosystem also features a lightweight, CPU-based rendering engine for medical images known as the Stone of Orthanc: This C++ toolkit is a building block to create heavyweight software, mobile applications (iOS or Android), or Web interfaces (through WebAssembly) that need to display or process medical images.

The Orthanc ecosystem is entirely free and open-source. The Orthanc server itself can be downloaded and used under the terms of the GPLv3 license. The official plugins are mostly released under the AGPLv3 license. The Orthanc ecosystem was designed to be as open and simple as possible, in order to cut down the DICOM learning curve by fostering compatibility between vendors and knowledge sharing about the DICOM standard, to the benefit of hospitals, research centers, public organizations, companies, or even general audience. Extensive technical documentation of the Orthanc ecosystem can be found in the Orthanc Book [13].

## Footnotes

1 In general, proposing Web interfaces instead of heavyweight client applications is important for hospital setups, as the installation of third-party software is often prevented for security, licensing, and maintenance reasons.

2 Note that the Orthanc server implements *disk space recycling*: The oldest series of images can be automatically deleted when the disk usage becomes higher than a predefined threshold, which is very handy to deploy such transient DICOM buffers.
